# Prospect and challenges of borophene bandgap formation: A comprehensive review

**DOI:** 10.1016/j.heliyon.2024.e36896

**Published:** 2024-08-24

**Authors:** Md. Abdullah, Mohammad Saidur Rahman, Mohammad Obayedullah, Sawda Ahmed Musfika

**Affiliations:** a Department of Mechanical Engineering, City University, Khagan, Savar, Dhaka, 1216, Bangladesh; b Department of Mechanical Engineering, Dhaka University of Engineering & Technology, Gazipur, 1707, Bangladesh; c Department of Civil Engineering, Bangabandhu Sheikh Mujibur Rahman Science and Technology University, Gopalgonj, 8100, Bangladesh; d Department of Civil Engineering, Khulna University of Engineering & Technology, Khulna, 9203, Bangladesh

**Keywords:** Borophene, Bandgap formation, Hexagonal lattice, Electronic properties, Materials science

## Abstract

A two-dimensional crystalline allotrope of boron, Borophene, has attracted much interest lately because of its unique electrical characteristics and possible uses in electronic devices. This thorough analysis examines the opportunities and difficulties related to the bandgap creation in boron, which is essential for its incorporation into semiconductor technologies. An introduction to the structural features of Borophene is given at the outset of the text, emphasizing its fascinating hexagonal lattice and tunable electronic properties. The review thoroughly explores the range of techniques used in synthesizing Borophene, covering both theoretical and experimental methods. It assesses how growth conditions, post-synthesis treatments, and substrate interactions affect the establishment of the bandgap in Borophene. The study also looks at how strain engineering, flaws, and impurities affect the bandgap, highlighting the necessity of exact control over these elements to get desirable electrical properties. We go into great length about the difficulties in Borophene bandgap engineering, including stability, scalability, and repeatability problems. The study critically evaluates the current body of knowledge, pointing out knowledge gaps and suggesting possible directions for further research. In addition, the paper discusses how external elements like humidity and temperature affect the stability of Borophene electrical characteristics, which complicates practical application. To sum up, this thorough analysis offers insightful information about the development of the borophene bandgap formation and a road map for scientists and engineers hoping to utilize borophene to its maximum potential in the future generation of electronic devices. The difficulties in synthesis and the complex interaction of several factors influencing bandgap creation highlight the necessity of ongoing multidisciplinary work to realize the technological potential of borophene.

## 1 Introduction

A monolayer of elemental boron makes up the two-dimensional (2D) substance known as boron. It possesses exceptional electrical, mechanical, chemical, optical, electronic, and catalytic qualities, among other unique physical, chemical, and electrical qualities [1]. Similar to graphene but with a different atomic configuration, Borophene possesses a hexagonal hole structure and a triangular lattice synergy [2]. Various methods, such as liquid phase exfoliation, molecular beam epitaxy, chemical vapor deposition, and ball milling, can create it [3,4]. Different morphological features of Borophene nanostructures, like thin and regular, few-layered flakes, can be produced using the synthesis procedures [5]. Borophene has demonstrated significant promise for use in supercapacitors, energy storage devices, sensors, and medical equipment. It is a desirable material for use in optoelectronics and energy storage applications due to its diverse electrical characteristics.

Supercapacitors, energy storage devices, sensors, and medical equipment can all benefit from Borophene exceptional electrical, optical, chemical, mechanical, and catalytic capabilities [2]. Experimental research indicates that techniques like liquid phase exfoliation, chemical vapor deposition (CVD), and beam epitaxy (MBE) enable the manufacture of benzophene [3]. Beyond the objectives the U.S. Department of Energy established, borobopene's moderate binding energy and reversible behavior make it a promising candidate for hydrogen storage [1]. Researchers can also use borophene with other materials, such as graphene, to form more stable heterostructures with improved electrical characteristics [6]. Borophene is a promising material for many fields because of its numerous features and applications.

Its optical clarity, strong heat conductivity, and great flexibility are all present [3]. Borobopene [2] demonstrates anisotropic plasmonics, high carrier mobility, and superconductivity. Other qualities include its ultrahigh heat conductivity and remarkable mechanical compliance [15]. Several techniques, such as liquid-phase exfoliation, sonochemical exfoliation, and the dissolution-segregation process in conjunction with chemical vapor deposition, have been used to manufacture borobopene [1,19]. It is possible to synthesize graphene/borophene heterostructures by placing graphene on top of the borophene layer. This creates a heterostack that can better maintain Borophene metallic nature and electrical characteristics. Borophene is a material with great potential for use in energy storage, sensors, and information storage.

Borophene heterostructures with improved stability and characteristics can be made by combining them with other materials, such as graphene [3]. Borophene has been investigated for usage in supercapacitors and batteries and hydrogen and oxygen evolution as energy applications [4]. Additionally, it has demonstrated promise in applications involving humidity and gas sensing [7]. However, researchers must overcome obstacles and conduct further studies to fully comprehend and realize the potential of borophene.

Due to its diverse electrical characteristics, beryllium is a desirable material for energy storage and optoelectronic applications [1]. Borophene is a promising building component for 2D electronics because of its remarkable mechanical and electrical qualities [2]. Researchers have discovered its distinct electrical, chemical, and physical characteristics, including its metallicity, transparency, conductivity, and chemical activity [3]. Borophene has been identified as a superior material [4]. Its electrical, optical, mechanical, chemical, and catalytic properties make it useful for supercapacitors, energy storage devices, sensors, and medical equipment. Borophene can potentially improve the characteristics of polymeric nanoarchitecture, which could result in a variety of multipurpose uses [7]. Borophene has also demonstrated potential as a lithium-ion battery electrode material because of its high specific capacity, outstanding rate capability, and cycle performance.

Borophene has a band gap of roughly 2.1 eV and is narrowly semiconductive [11]. These properties make it good for high-speed transistors, field emission, and optoelectronic detection [1]. They are made possible by its large specific surface area, high conductivity, and trim work function. Researchers have synthesized graphene/borophene heterostructures using borophene, demonstrating an improved ability to retain the metallic character and electrical characteristics of borophene [19]. Energy transitions at high-symmetry locations cause peaks in borophene's optical conductivity [20]. For borophene to be used in semiconductor devices, it must have a bandgap [8,9]. Two-dimensional borobopene has unique characteristics, such as thermal solid conductivity and electron mobility [10]. Its tunable bandgap makes borophene suitable for use in optoelectronic devices and sensors [11]. The most efficient forms of deformation for widening the band structure's energy gap have been investigated in relation to various van der Waals heterostructure combinations, including borobopene [12]. Hydroxy-functionalized borobopene's ability to be experimentally synthesized and its adjustable band gap make it suitable for developing photoelectrochemical photodetectors with increased photocurrent density and photoresponsivity. Low-pressure chemical vapor deposition forms borophene sheets with a narrow semiconductive character, indicating potential use in high-performance nanodevices. Theoretical calculations using ab initio quantum techniques [12] reveal Borophene apparent bandgap in certain regions of the electronic band structure.

Bandgap development is an important factor influencing borobopene's electrical and optical properties. The size of the bandgap determines the material's conductivity and its ability to both absorb and emit light. Numerous research studies have examined the impact of various conditions on the creation of bandgaps in Borophene. For instance, bilayer Borophene rotation orientations can change the energy band topologies and narrow the band gaps [8]. Furthermore, mechanical alterations, such as compression or stretching, can adjust the electrical characteristics of van der Waals heterostructures based on borobopene [13]. Bandgap is also influenced by the fabrication of freestanding Borophene nanosheets; in contrast to boron powders, an increase in bandgap is caused by quantum confinement effects [11]. These findings emphasize how critical it is to understand and manage bandgap development in borobopene in order to use it in future optoelectronic and electrical devices.

Borophene's bandgap development is controllable in a number of ways. One strategy is to modify the bilayer Borophene's rotation angles, which can shift the energy band structures and shrink the band gap sizes [13]. An additional technique is applying mechanical deformations, such as stretching or compression, to adjust the electrical characteristics of van der Waals heterostructures based on borobopene and create energy gaps in the band structure [8]. By altering the thickness of borobopene through liquid-phase exfoliation, researchers can adjust its band gap, resulting in semiconducting borobopene with a customizable band gap [10]. Borophene may also show energy gaps in the band structure when it comes into contact with other substances, like boron nitride or molybdenum disulfide [9]. All things considered, these results offer an understanding of the exact regulation of bandgap creation in borobopene for prospective uses in optoelectronics and nanoelectronics [14].

Emerging as a two-dimensional nanoelemental material, borobopene exhibits potential uses in energy harvesting, sensors, energy storage, energy conversion, and information storage [15]. Anisotropic plasmonics, high carrier mobility, mechanical compliance, optical transparency, ultrahigh thermal conductance, and superconductivity are only a few of its unique qualities [16]. Supercapacitors, batteries, hydropower generators, humidity sensors, gas sensors, pressure sensors, and memory are just a few applications for borobopene [17]. It can be used as a photothermal therapeutic in the treatment of tumors because it also exhibits a high near-IR light-induced photothermal effect [18]. Researchers have produced and employed borophene nanosheets in nonvolatile memory devices due to their good rewriteable nonvolatile memory performance and stability [9]. Because of its unique chemical binding and metallic properties, borobopene is a good choice for sensors, excitonic devices, and flexible hetero-layered electronics. Researchers have looked at how Borophene interacts with other materials and found clear peaks in the electronic density of states and differential current signals. These show that quantum states can interact with each other at the interface. Researchers have employed heterolayered stacks based on borobopene as molecular anchoring platforms in molecular detection [9].

## 2 Theoretical insights into borophene bandgap formation

**Fig. 1:**
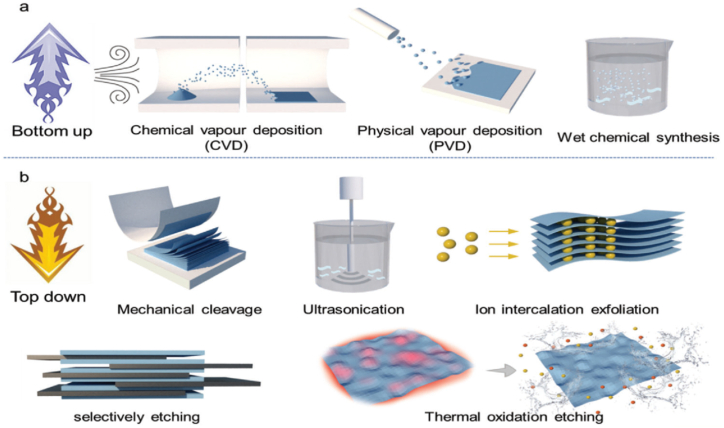
**(a, b):** The primary technique for creating 2D nanosheets. a) Bottom-up approach: wet chemical, CVD, and PVD [22]. b) Top-down technique: thermal oxidation etching, ion intercalation exfoliation, mechanical cleavage, and ultrasonication [22].

Theoretical research has been done to comprehend the electrical characteristics of borophene and its possible uses [12,21]. In one study, borophene and other 2D materials, including zinc oxide (ZnO) and gallium nitride (GaN), were employed to create van der Waals heterostructures [22,23]. Another study created hydroxy-functionalized borophene with different thicknesses and used those results to experimentally validate the tunability of borophene's band gap. Compared to other 2D materials, borophene-OH demonstrated improved photocurrent density and photoresponsivity in photoelectrochemical (PEC) photodetectors. These results show the promise of borophene for optoelectronic applications by offering theoretical insights into the creation and tunability of its bandgap. Fig. 1(a) shows the primary technique for creating 2D nanosheets. a) Bottom-up approach: wet chemical, CVD, and PVD [22]. b) Top-down technique: thermal oxidation etching, ion intercalation exfoliation, mechanical cleavage, and ultrasonication [22].

Theoretical insights into borophene bandgap creation can lead to the development of novel materials by understanding its structural and electrical characteristics and interactions with other materials. First-principles calculations based on density functional theory (DFT) can provide accurate information on the band structure and density of states (DOS) of borophene [24]. These calculations can identify the most effective deformation patterns for creating a gap in energy within the band configuration of substance heterostructures [8]. Also, adding oxygen can change the way borophene connects to a metal base, which lets the electrical properties of borophene sheets be customized [12]. Designers can create one-dimensional nanowires with various electrical characteristics using the inverse sandwich structure of boron nanowires, providing new possibilities for making boron-based nanowires [23]. These theoretical understandings can guide the creation of novel materials with specific band structures and electrical characteristics. Theoretical models and computational simulations predict electronic features, specifically the generation of band gaps, for semiconductors and other materials. Understanding how these materials behave and function depends on these expectations.

**Fig. 2:**
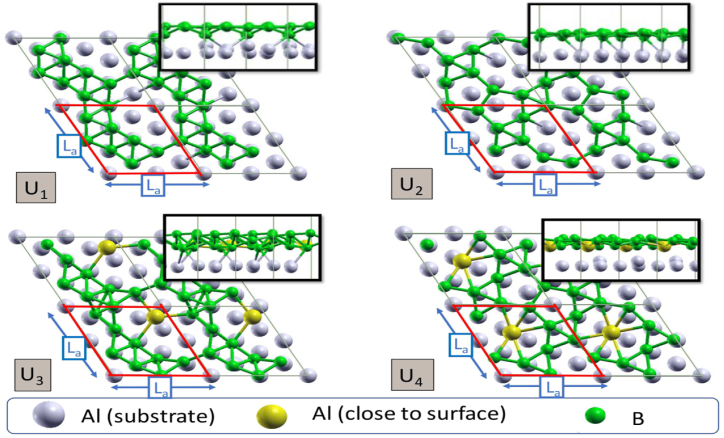
The U1, U2, U3, and U4 polymorphs of borophene in top and side views. The increased electronegativity of boron (with a length scale of La = 5.71 °A) is thought to create the gold color of aluminum to indicate that the aluminum atoms are separated from the substrate and tend to adsorb by surface. The red hexagon represents the conventional cell of each polymorph [23].

A study [25] suggested a modified Behler-Parrinello architecture that uses machine learning approaches to predict band gaps from density functional theory calculations. A separate study discovered new materials with exceptional features by predicting quaternary semiconductor band gaps using machine learning and DFT computations [26]. Fig. 2 shows the U1, U2, U3, and U4 polymorphs of borophene in top and side views. The increased electronegativity of boron (with a length scale of La = 5.71 °A) is thought to create the gold color of aluminum to indicate that the aluminum atoms are separated from the substrate and tend to adsorb by surface. The red hexagon represents the conventional cell of each polymorph [23].

**Fig. 3:**
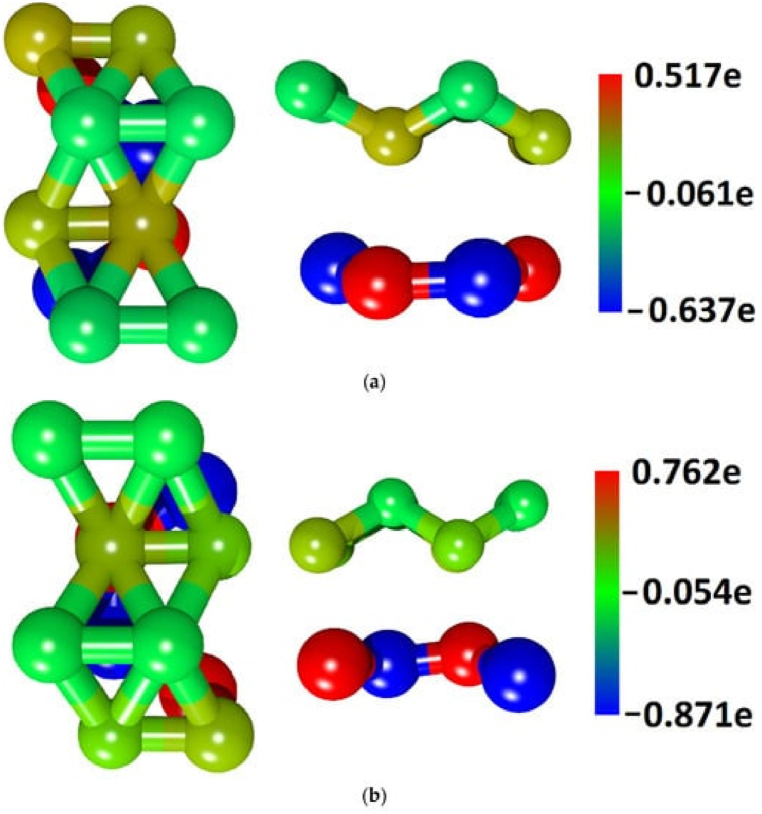
**(a, b):** The distribution of electron charge density over the atoms in the van der Waals heterostructures' supercells, which contain borophene/GaN [8] (a) and borophene/ZnO (b) [8].

More research looked at the electronic properties of broad bandgap materials using density functional theory calculations. This gave scientists new information about the electronic properties of alloys, how heat moves, and how phonons limit electron movement [27]. One study, which combined computational and experimental data sources, demonstrated how ensemble learning can effectively model experimental data for band gap prediction [28]. Fig. 3 (a, b) shows the distribution of electron charge density over the atoms in the van der Waals heterostructures' supercells, which contain borophene/GaN [8] (a) and borophene/ZnO (b) [8].

There are several drawbacks to using theoretical models and computational simulations to forecast the electrical characteristics of bandgap development. First, traditional methods for studying the structure of electronics that are based on density functional theory (DFT) are not very accurate and take a long time to run [29].

This is because they use exchange-correlation functional approximations. More accurate methods like GW and hybrid functionals require a lot of computing power and aren't good for high-throughput screening. On the other hand, standard DFT approximations can be very wrong about band gaps [26]. Due to the band gap problem in local density approximation (LDA) and generalized gradient approximation (GGA) [30], DFT always gets the band gaps of insulating systems wrong. Machine learning (ML) techniques anticipate band gaps to circumvent these restrictions, providing a faster and more precise solution [31,32]. Theoretical models and computational simulations predict the electrical characteristics of bandgap generation. Accurate band-gap predictions for various materials have been made using machine learning techniques like random forest regression and neural network models [[33], [34], [35], [36]]. These models can overcome the drawbacks of traditional density functional theory (DFT)-based electronic structure approaches, which include large processing costs and poor accuracy [22]. Machine learning algorithms may accurately predict band gaps by combining physical data like electronegativity, superconductivity temperature, and bulk modulus. Machine learning models based on calculations from density functional theory can greatly decrease the computational cost compared to hybrid functionals. These theoretical models and computational simulations make the identification and creation of functional materials possible, offering a quick and effective method for evaluating and projecting material band gaps [37]. Table 1 provides an overview of recent work on the theoretical understanding of creating borophene bandgaps.

**Table 1:** An overview of recent work on the theoretical understanding of creating borophene bandgaps.

SL	Year		Reference
1	March 2023	It was possible to form distinct B5 clusters on monolayer borophene (MLB). In a periodic pattern, B5 clusters bind to specific MLB sites.	[92]
2	May 2022	The chemical binding of borophene is unusual, and its thermal conductivity is high. Heterolayered borophene stacks have applications in molecular and excitonic sensing.	[93]
3	May 2023	The borophene bilayer (BBL)/m-surf interface on metal surfaces (Ag, Au, and Al) reveals metalization and ohmic connections with a Schottky barrier.	[94]
4	November 2021	The two-dimensional substance borophene has distinct characteristics and potential applications. This work covers the synthesis, characteristics, and potential uses of borophene.	[95]
5	January 2021	Borophene refers to two-dimensional boron phases with various structural changes. Their structure and stability are better understood using electron counting techniques and theoretical developments.	[96]
6	August 2020	DFT calculations demonstrate the formation of stable borophene superlattices through the band engineering of a zigzag nanoribbon-based borophene superlattice.	[97]
7	November 2021	One studies the adsorption of oxygen atoms on borophene doped with aluminum, argentum, and aurum. Adsorbed O atoms determine the electronic and optical characteristics of metal-doping borophene.	[98]
8	March 2019	On Au (111) substrates, borobopene is synthesized. As boron diffuses into Au and separates from the surface, borophene islands are created.	[99]
9	December 2017	Borophene's s-p orbital hybridization gives rise to its distinctive metallic characteristics. Defective engineering and chemical functionalization do not cause the band gap in borophene to open.	[100]
10	October 2017	Calculations based on fundamental principles investigate the engineering of W-borophene band structure. In W-borophene, shear strain can cause the band gap to expand.	[101]

Numerous studies have looked into how structure, strain, and doping affect bandgap tunability. Fe-doped lithium niobate experienced a significant reduction in bandgap when subjected to compressive and tensile strains, with the most pronounced decrease observed at 20 % tensile strain [33]. It was discovered that biaxial strain, with the bandgap growing under tensile strain and decreasing under compressive strain, increased the absorbance of APbI3 (A = Rb and Cs) perovskites in both the visible and ultraviolet light energy ranges [34]. The hybridization of the Zn3d and N2p orbits in ZnGa2O4 films, caused by nitrogen doping, helps reduce the optical band gap [35]. Arsenic substitution for black phosphorus reduced the bandgap and enhanced air stability, making it suitable for ambient-stable photodetectors and optical modulators [4]. Biaxial strain impacts the bandgap and mechanical characteristics of CsGeCl3 perovskites, qualifying them for solar photovoltaic applications [36].

Theoretical frameworks guide experimental design for specific bandgap engineering [[38], [39], [40]]. Using computational techniques such as density functional theory, these frameworks anticipate the bandgap as a function of composition and strain [41,42]. It is possible to make precise bandgap predictions using specific density functionals and computational procedures. Researchers have used these frameworks to engineer the breadth and suppression of bandgaps and identify the most promising materials for device design, including phononic crystals and III-V semiconductors. Ab initio computations and machine learning approaches have integrated to direct strain engineering and develop material performance and properties. These theoretical frameworks enable focused and practical material synthesis and optimization, providing experimentalists in bandgap engineering with valuable information [42].

In particular, creating new theoretical frameworks to direct experimental design in bandgap engineering has a number of possible advantages. First, these frameworks can help improve the design of complicated nonlinear structures, like the main resonance frequency and damping efficiency of an electricity distribution system (EDS) [38]. Two-dimensional (2D) materials can tune bandgaps, which is essential for their potential applications in spintronics, optoelectronics, and nanoelectronics [43]. Furthermore, these frameworks have the potential to help create desired gap widths and suppression levels by optimizing the band structure of three-dimensional phononic crystals [44]. Additionally, they can support the growth of anti-deficit strategies in engineering education research by directing study designs using crucial theoretical frameworks for emancipation [41]. These new theoretical frameworks can enhance bandgap engineering to be more practical, efficient, and effective in various engineering applications.

## 3 Experimental approaches in synthesizing borophene with tailored bandgap

Recent work has focused on experimental methods for producing borophene with a customized bandgap. Researchers have investigated numerous techniques, including density functional theory-based first-principles computations [45], liquid phase exfoliation [2], and experimental synthesis on various substrates [15]. Liquid-phase exfoliation has shown promise as a scalable technique for producing borophene [13]. Multiple borophene growth methods and architectures have been investigated through experimental synthesis on various substrates [11]. Researchers studied bilayer borophene with varying rotation angles structurally and electrically through first-principles computations. These methods have advanced our understanding of borophene's characteristics and may assist us in modifying its bandgap for specific applications.

**Fig. 4:**
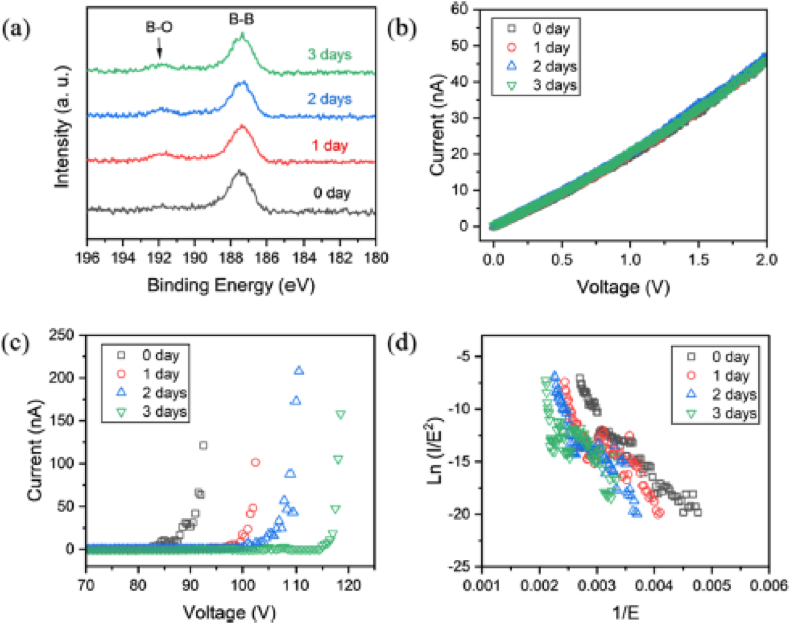
**(a**–**d):** (a) The borophene sheets' XPS spectrum after varying air exposure times [11]. The I-V and FE curves of individual borophene sheets at varying exposure durations are shown in (b, c) [11]. (d) The FN plots that correspond to them [11].

One method for creating borophene with a certain bandgap is chemical vapor deposition (CVD), which shows great promise. Two-dimensional materials like borophene have unique qualities like low density, strong chemical stability, and high electron mobility [2]. Experimental research has demonstrated that CVD techniques, such as molecular beam epitaxy (MBE) and low-pressure CVD (LPCVD), can be used to synthesize borophene [1,46]. High-ambient-stability 2D tetragonal borophene sheets have been successfully fabricated via LPCVD [47]. Another approach that has been suggested as a viable one for increasing the synthesis of borophene is the liquid-phase exfoliation method [11]. Researchers have investigated modified Hummers' method and sonochemical exfoliation for producing freestanding borophene. These ways of making borophene let you make it with a specific bandgap, which is important for its use in field emission, optoelectronic detection, and fast transistors. Fig. 4(a–d) shows (a) the borophene sheets' XPS spectrum after varying air exposure times [11]. The I-V and FE curves of individual borophene sheets at varying exposure durations are shown in (b, c) [11]. (d) The FN plots that correspond to them [11].

**Fig. 5:**
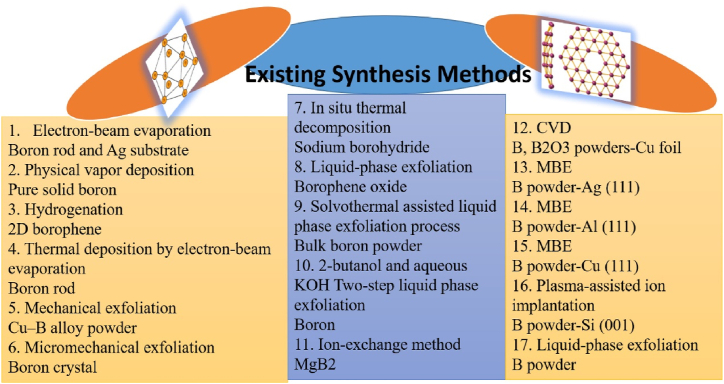
A summary of the current borophene manufacturing process [49].

Researchers synthesized bandgap-tailored borophene using chemical vapor deposition (CVD) methods. Researchers effectively used a low-pressure CVD technique to create 2D tetragonal borophene sheets [17]. Researchers produced high-quality freestanding α-rhombohedral borophene nanosheets using a probe ultrasonic technique in several organic solvents [48]. Another study looked at various ways to make borophene nanostructures, such as CVD methods [3]. They also used a two-zone CVD method to build structurally stable few-layer β12-borophene on copper foils. A study that used an electrochemical exfoliation process presented a unique way to synthesize borophene. These studies show how flexible CVD methods are for making borophene with specific bandgaps, which means it could be used in high-end electronics and electronics that use light [49]. Fig. 5 summarizes of the current borophene manufacturing process [49].

**Fig. 6:**
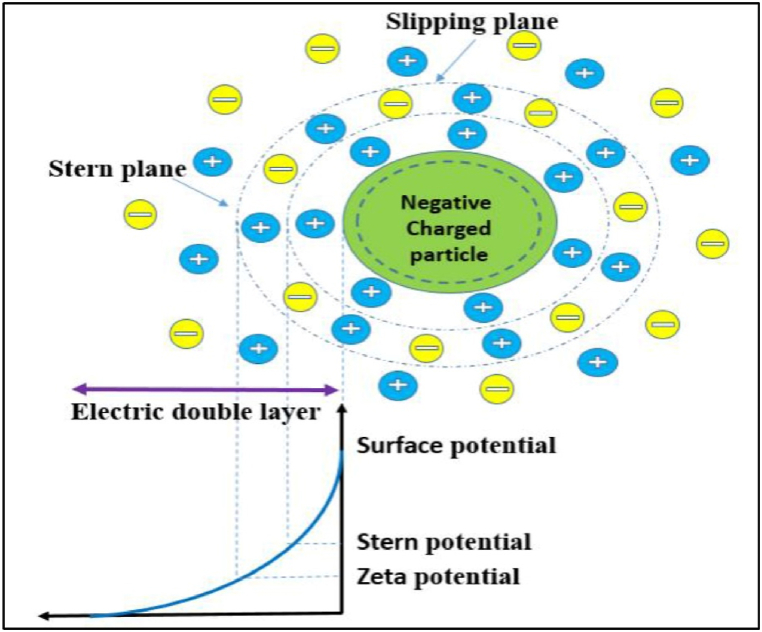
Schematic of a zeta-potential block for surface charge [49].

Chemical vapor deposition (CVD) is a flexible method for synthesizing borophene with various benefits. It makes it possible to produce high-quality, large-area films with adjustable thickness [11]. CVD is a popular technique for producing borophene that uses copper foils as a substrate [1]. Borophene created by CVD exhibits excellent electrical transport and field emission qualities, even after being exposed to air for a few days [50]. High-crystalline and chemical-grade graphene/borophene heterostructures can be synthesized by combining the dissolution-segregation technique with CVD [2]. These heterostructures can better maintain borophene's electrical characteristics and metallic nature [48]. Nevertheless, pristine borophene is vulnerable to oxidation, which can reduce its stability and potential uses. This is one of CVD's drawbacks. The primary methods for synthesizing borophene are chemical vapor deposition (CVD) and molecular beam epitaxy (MBE) [2]. Borophene synthesis can utilize these techniques due to its complex chemical connections and reactivity [51]. Researchers produced tetragonal borophene sheets with good ambient stability using low-pressure chemical vapor deposition (LPCVD) [11]. van der Waals epitaxy has also synthesized borophene on nonmetallic substrates [19]. These synthesis methods tailor borophene's bandgap, making it appropriate for a range of uses in high-performance electrical and optoelectronic devices [52]. Fig. 6 displays the schematic of a zeta-potential block for surface charge [49].

In the field, producing high-quality, large-area borophene has proven difficult. One method uses chemical vapor deposition to synthesize multilayer hexagonal boron nitride (hBN) at the centimeter scale on iron-nickel alloy foil [53]. An alternative technique entails creating millimeter-sized borophene sheets in an ultrahigh vacuum on an Ir (111) surface, which are then successfully transferred to a Si wafer via electrochemical delamination [54]. Researchers have also developed an innovative technique that transforms boron into borophene at high temperatures by utilizing an electrochemical exfoliation process and a boron cathode [55]. Boron synthesis as large-area single-crystal surface structures on a Cu (111) substrate has produced two-dimensional copper boride instead of borophene [56,57]. These studies show many methods and strategies, each with pros and cons, for creating high-quality, large-area borophene.

**Fig. 7:**
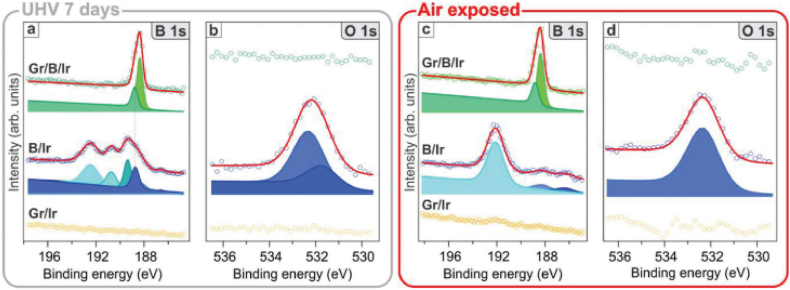
**(a**–**d):** Evaluation of B 1s and O 1s emissions determined on two distinct regions after seven days in the ultraviolet (UHV) laboratory (a and b on the left panel) [1] and after 90 min of exposure to the air (c and d on the right panel) [1].

Many studies have explored controlling structural phases to achieve desirable band gaps. One method is to create a bandgap gradient by applying elastic deformation to adjust a material's physical properties locally and reversibly [58]. Gas-phase cation exchange (GCE) is another way to make transition metal sulfides (TMSs) with specific bandgaps and known crystal forms [59]. This is done by causing a certain topological transformation (TT). Exfoliate transition metal dichalcogenides (TMDs) have also demonstrated potential for producing bandgaps with spatial texture and strain-tunable characteristics [60]. Low-frequency bandgaps have been constructed in vibration isolation using meta structures made of composite-compliant bistable units with geometric nonlinearity [61]. Adding a layer in the middle with a variable refractive index and a simple structure design using a 1D photonic crystal to control the bandgap and bandgap window has also been suggested [62]. Fig. 7(a–d) shows the contact integrity in air and vacuum exposure as well as an illustration of the B 1s and O 1s wavelengths measured in three different positions: two weeks into the ultraviolet (UHV) container (a and b on the left panel) [1] and 90 min into the air (c and d on the right panel) [1].

## 4 Challenges and opportunities in characterizing borophene bandgap

**Fig. 8:**
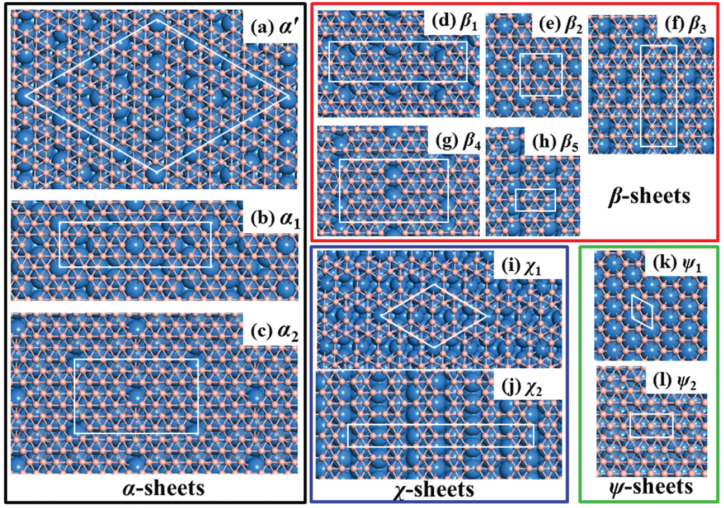
**(a**–**l)**: The silver (111) surface's prospective substance atomic structures [65]. Examining the four categories of borophenes: sheets with α, β, χ, and ψ [65]. The silver (111) substrates display the borophene unit cell in solid outlines [65]. The B and Ag atoms are blue and brick red, respectively [65].

Determining the borophene's bandgap presents both opportunities and challenges. Borophene, a two-dimensional material containing boron, has demonstrated significant potential for energy storage devices, sensors, and optoelectronics [3,55]. However, characterization is a complex problem because borophene's production and experimental properties sometimes match theoretical expectations [8]. Nevertheless, there have been developments in borophene synthesis, such as the creation of efficient manipulation tools and large-scale synthesis methods [54]. Researchers have also looked into how mechanical deformations change the electronic structure of borophene to make it behave differently and create an energy gap in the band structure [6]. More investigation is required to fully realize the promise of borophene for various applications and overcome the difficulties in describing its bandgap. Several investigations used scanning tunneling microscopy (STM) to characterize the bandgap of borophene. Molecular beam epitaxy (MBE) growth successfully generated honeycomb borophene on an Al (111) substrate. The STM pictures they took showed a perfect single layer of borophene with a honeycomb structure that looked like graphene and wasn't buckled [63]. They used liquid-phase exfoliation to create hydroxy-functionalized borophene (borophene-OH), and by adjusting its thickness, they demonstrated an adjustable band gap. Additionally, they demonstrated improved photocurrent density and photoresponsivity by creating functional electrodes for photoelectrochemical (PEC) photodetectors using borophene-OH [64]. The silver (111) surface's prospective substance atomic structures are shown in Fig. 8(a–l) [65]. Examining the four categories of Borophene: sheets with α, β, χ, and ψ [65]. The silver (111) substrates display the borophene unit cell in solid outlines [65]. The B and Ag atoms are blue and brick red, respectively [65].

**Fig. 9:**
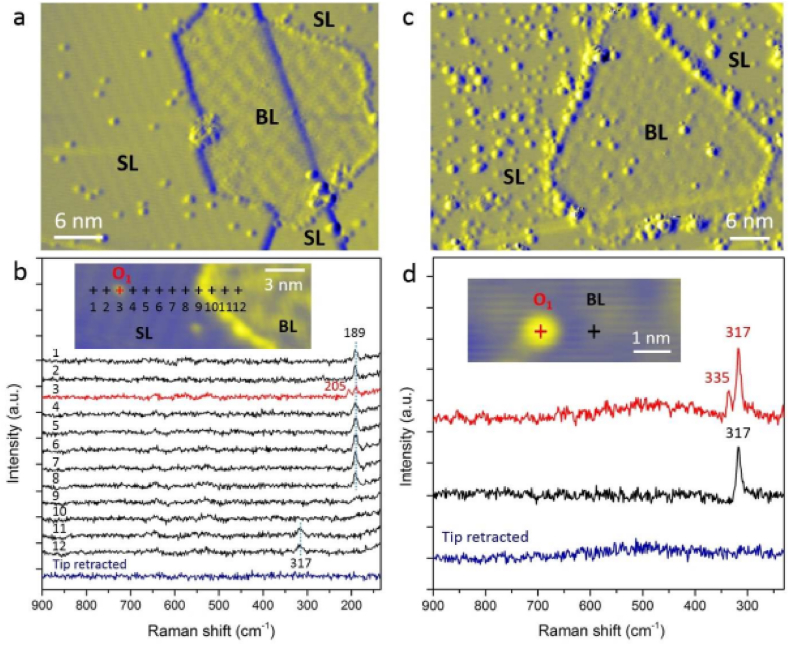
**(a**–**d):** Chemical discoveries from the atomic scale on mixed-dimensional borophene oxidation. a) Topographic derivative STM image of borophenes SL and BL after exposure to a low O_2_ dose (300 L) at ultrahigh vacuum [66]. b) A TERS line scan of an oxygen adatom on SL borophene follows the tip trace in the inset STM image [66]. c) Topographic derivative STM image of the borophene species SL and BL following exposure to an elevated O2 dose (1800 L) [66]. d) The STM image inset displays the oxidized BL borophene TERS spectra. Tunneling conditions: 1.0 V at 55 pA, 1.2 V at 100 pA, 0.7 V at 60 pA, and 0.7 V at 300 pA (a, b, c) [66]. TERS parameters: (d) 200 mV, 1 nA, 30 s; (b) 100 mV, 1 nA, 5 s, each point with a step length of 1 nm [66].

Additionally, they used first-principles calculations to study borophene's atomic structure and electrical characteristics on an Ag (111) surface. The stable borophene structures showed average metallic conductivity [10]. A different study found that controlled oxidation of borophene in ultrahigh vacuum (UHV) leads to single-atom covalent alteration of the borophene basal plane [65]. Fig. 9(a–d) shows chemical discoveries from the atomic scale on mixed-dimensional borophene oxidation. a) Topographic derivative STM image of Borophene SL and BL after exposure to a low O2 dose (300 L) at ultrahigh vacuum [66]. b) A TERS line scan of an oxygen adatom on SL borophene follows the tip trace in the inset STM image [66]. c) Topographic derivative STM image of the borophene species SL and BL following exposure to an elevated O2 dose (1800 L) [66]. d) The STM image inset displays the oxidized BL borophene TERS spectra [66]. Tunneling conditions: 1.0 V at 55 pA, 1.2 V at 100 pA, 0.7 V at 60 pA, and 0.7 V at 300 pA (a, b, c) [66]. TERS parameters: (d) 200 mV, 1 nA, 30 s; (b) 100 mV, 1 nA, 5 s, each point with a step length of 1 nm [66].

UHV-TERS validates the three-dimensional lattice structure of borophene and shows that bilayer borophene has better chemical stability than monolayer borophene by offering atomic-level chemical insight into the vibrational fingerprint of borophene [[66], [67], [68]]. Table 2 compares the computed values, cm−1, and the TERS phases during the α stage [69].

**Table 2:** Comparison the computed values, cm−1, and the TERS phases during the α stage [69].

TERS	Simulation	Modes
116.8	107.85	E7E7
157.3	188.6	E6E6
339.0	352.94	A41A14
406.4	425.2	A31A13
446.8	507.83	A32A23
702.6	684.32	E4E4
920.4	907.16	E3E3
1230.0	1199.10	Vibration of SC

One study characterized BL borophene using UHV-TERS, demonstrating its superior chemical stability compared to monolayer borophene [68]. Another study that used UHV-TERS assessed the interfacial interactions of heterostructures based on borophene and observed subtle ripples and compressive strains in the borophene lattice [67]. They investigated the local vibrational characteristics of α-borophene using tip-enhanced Raman spectroscopy (TERS). They discovered an odd amplification of high-frequency Raman peaks that was connected to its distinct buckling on the Ag (111) surface [69]. Researchers have demonstrated that Raman spectroscopy, particularly TERS and UHV-TERS, is a suitable experimental method for determining borophene's bandgap and other characteristics.

**Fig. 10:**
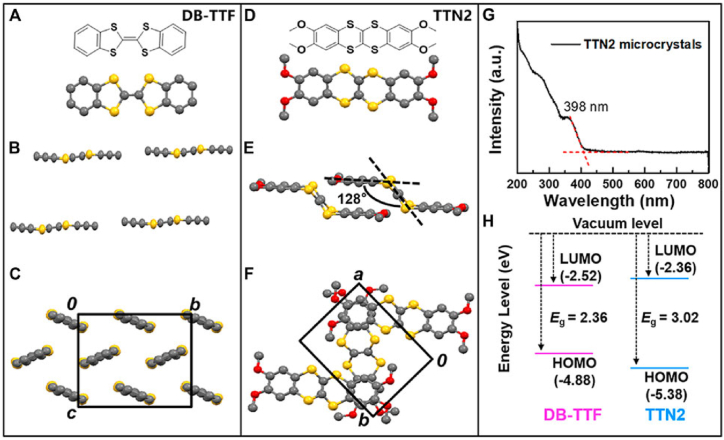
(A–H): The molecular makeup and packing patterns of ttn2 microcrystals (h) and db-ttf and ttn2 microcrystals (a–c) and (d–f), respectively [74]. Bandgaps and energy levels of ttn2 and db-ttf [74].

Numerous studies have addressed issues of resolution and repeatability in bandgap measurement. Unlike typical bandgap references, one study developed a programmable high-precision bandgap reference that can match the accuracy needs of all technological corners [70]. Another article addressed the problems with reproducibility and offered solutions, highlighting the value of measurement science in overcoming these problems [71]. These difficulties were demonstrated by a significant interlaboratory comparison of the mass fraction measurement, inconsistent Hubble-Lemaitre constant measurement results, and the non-reproducible mass fraction of arsenic in Kudzu measurements [72]. These studies show the importance of precise and repeatable bandgap measurements and offer suggestions for overcoming related difficulties. It is essential to comprehend how environmental interactions affect bandgap stability in order to design stable semiconductors for various applications. Numerous studies have looked into how different ecological conditions affect bandgap stability. Exposure to oxygen, high humidity, or high temperature significantly accelerated the breakdown of narrow-bandgap hybrid organic-inorganic mixed (Sn + Pb) perovskites [73]. However, as evidenced by the high stability of OSCs based on 2,3,8,9-tetramethoxy [1,4] benzodithiino [2,3] benzodithiine (TTN2) [74], controlling the molecular configuration of OSCs is an effective strategy to achieve both environmental and operational stability. Furthermore, it was discovered that creating metal cation-alloy perovskites (MABaxPb1–xI3) improved stability and permitted customized bandgaps [75]. Research on the role of surface charge in material stability has also demonstrated that the surface's ability to draw reactive ions can impact semiconductor stability [76]. These investigations offer critical new understandings of the methods and approaches for attaining bandgap stability under various environmental circumstances. Fig. 10(A–H) shows the molecular makeup and packing patterns of TTN2 microcrystals (H) and DB-TTF and TTN2 microcrystals (A–C) and (D–F), respectively [74]. Bandgaps and energy levels of TTN2 and DB-TTF [74].

The interactions between its surroundings and bandgap stability significantly impact a semiconductor's performance and longevity. For instance, research was done on the deterioration of narrow-bandgap hybrid organic-inorganic mixed tin (Sn) and lead (Pb) perovskites in various environmental settings [73]. It was discovered that exposure to high temperatures or high humidity alone had less effect than exposure to oxygen, which caused the most severe deterioration [77]. The interface between the semiconductor and the environment significantly influences the stability of organic field-effect transistors (OFETs) [78]. OFET-based chemical sensors can enhance their sensing performance by employing chiral sensing layers and optimizing the device structure [74]. The band structure of semiconductors can also be impacted by the interaction of electrons with ambient disturbances, such as phonons or impurity atoms [79]. Adjusting the molecular structure of organic semiconductors can tune their bandgaps and increase their environmental stability. In humid environments, surface charge affects semiconductor stability; positively charged surfaces are less stable than negatively charged surfaces.

## 5 Prospects for borophene bandgap engineering in electronic applications

Researchers experimentally produced borophene sheets with adjustable band gaps between 0.65 and 2.10 eV using liquid-phase exfoliation [11]. Researchers have discovered that these borophene sheets exhibit electrical solid conductivity, good ambient stability, and a narrow semiconductive nature [10]. Borobophene is a promising material for optoelectronic detection, field emission applications, and high-speed transistors due to its distinct shape and electrical properties [6]. Beyond the targets stated by the U.S. Department of Energy, borophene's modest binding energy and reversible behavior make it a promising material for intermediate hydrogen storage [45]. Borophene presents intriguing prospects for bandgap engineering and has the potential to be used in a range of electrical applications.

Composed of atoms of boron, borophene has demonstrated considerable promise for a range of electronic applications, including optoelectronics, sensors, and transistors [1]. It is a strong contender for these applications due to its remarkable qualities, which include outstanding electrical, optical, chemical, mechanical, and catalytic properties [3]. Borophene exhibits distinct crystallographic structural phases, great flexibility, high heat conductivity, and metallic properties [17]. Various methods, such as chemical vapor deposition and ultrasonic methods [9,19], have synthesized borophene to produce superior freestanding nanosheets. It has also been possible to synthesize graphene/borophene heterostructures, which permit borophene to maintain its electrical characteristics and metallic nature even in a reactive environment. These advances in borophene research enable the creation of high-performance electronic devices and bandgap engineering. Table 3 presents a summary of current research on the potential applications of borophene bandgap engineering in electronics.

**Table 3:** A summary of current research on the potential applications of borophene bandgap engineering in electronics.

SL	Year		References
1	October 2019	Integrating graphene with borobopene creates 2D heterostructures. It is possible to integrate graphene with borophene both vertically and laterally.	[90]
2	August 2020	Borophene is a potential 2D material with distinct structural and electrical properties. Researchers explore various methods of developing and potential uses for borophene.	[86]
3	January 2021	Researchers have experimentally produced multiple polymorphs of borobopene. Superconductivity and massless Dirac fermions are two unusual characteristics of borobopene.	[91]
4	May 2021	This research examines the synthesis, characteristics, and uses of borophene. Supercapacitors, batteries, hydrogen storage, and medicinal applications are among the potential uses for borobopene.	[84]
5	October 2021	A prospective two-dimensional polymer for efficient gas identification is borophene. Benzophenone-based sensors provide good selectivity, sensitivity, and fast response times.	[85]
6	February 2022	In the presence of air, borophene breaks down instantly. In UHV, there is controlled oxidation upon exposure to molecular oxygen.	[83]
7	June 2022	Oxygen doping decreases the charge transfer between aluminum and borophene. The thermodynamic stability of the Al (111)-oxygenated borophene contact is demonstrated.	[82]
8	September 2022	Researchers have demonstrated the atomistic models of the vertical heterostructures of borophene/GaN and borophene/ZnO. These heterostructures feature a gapless band structure and are energetically stable.	[87]
9	March 2023	Researchers make borophane via chemical vapor deposition on copper foils. Borophane has promising properties for nanophotonic devices and photoelectric solid reactions.	[88]
10	May 2023	The 12-B/Gr heterostructure's electrical and structural characteristics were examined, showing excellent conductivity for possible uses.	[89]

Borophene's unique qualities make it suitable for bandgap engineering in electrical applications [55]. Outstanding properties of borophene include low density, high electron mobility, and strong chemical stability when compared to other 2D materials [3]. It exhibits a band gap of roughly 2.1 eV and is narrowly semiconductive [13]. Borophene may be more advantageous than metal-based complex hydrides for hydrogen storage, perhaps exceeding DOE targets [6]. It currently requires more work to develop both practical and profitable borophene-based solid-state storage solutions [11]. Liquid-phase exfoliation has demonstrated potential as a production process for producing borophene sheets on a larger scale. Borophene is a promising material for electrical applications because of its unique qualities and potential for bandgap engineering; nonetheless, further study is required to overcome its drawbacks and make practical use possible.

It is possible to engineer borophene's bandgap to enhance its functionality in electronic applications. Twisted bilayer borophene can lower band gap widths and change the energy band topologies at different rotation angles [2]. Oxygen doping is an additional technique that can alter the strength and electrical properties of borophenes by reducing the charge transfer between them and the substrate [1]. Mechanical deformations such as stretching or compression can adjust the electrical structure of borophene by creating an energy gap in the band structure [11]. These techniques offer ways of accurately regulating borophene's bandgap, improving its suitability for various electronic uses.

One of the difficulties in utilizing borophene in electronic applications is the requirement for solid-state storage materials for hydrogen storage that are both effective and affordable [13]. Furthermore, borophene synthesis remains a challenging problem since it needs to meet theoretical assumptions at the appropriate level [23]. High-performance electronic and optoelectronic devices find it difficult to employ borophene extensively due to its structural instability and requirement for a metal substrate during deposition [3]. Also, the current state of research shows that a lot of work needs to be put into developing borophene-based materials for sensors, energy storage, information storage, energy conversion, and energy harvesting [6]. Even with its potential, borophene is currently impractical for industrial use; more research is required to overcome these obstacles and realize borophene's full potential in electronic applications.

For bandgap engineering in electronic applications, borophene has potential. Researchers have studied several vans der Waals heterostructure topologies using borophene and other two-dimensional materials [15]. Mechanical deformations such as stretching and compression can successfully adjust the electrical characteristics of heterostructures based on borophene, leading to the appearance of energy gaps in the band structure [55]. Researchers have created borophene-OH, a hydroxy-functionalized version of borophene with an adjustable band gap achieved by altering its thickness [8]. Researchers have discovered that borophene exhibits distinct electronic signatures of interfacial coupling with other materials [80]. Researchers can use these signatures for molecular detection and programmable functioning. Studies have demonstrated that borophene's low-loss surface plasmon polariton (SPP) characteristics and its ability to generate and transport plasmon-driven hot carriers make it suitable for applications in photocatalytic reactors and next-generation optoelectronic devices [81]. The semi-hydrogenated form of borophene, called α′-4H borophene, is a good candidate for flexible optoelectronic uses because it has strong excitonic states and a variable band gap.

## 6 Conclusion

To sum up, the possibilities and difficulties surrounding the production of the borophene bandgap constitute a dynamic and developing field in materials science and nanotechnology. Borophene's distinct structural and electrical characteristics present intriguing prospects for its use in semiconductor devices; nonetheless, achieving its maximum potential requires overcoming several severe obstacles. The thorough review has offered a detailed analysis of the several approaches to synthesizing borophene, highlighting the importance of growth factors, post-synthesis treatments, and substrate interactions in adjusting the bandgap. However, attaining stability, scalability, and reproducibility in the synthesis processes is a challenge that calls for coordinated efforts from the scientific community. Extensive research has been conducted on the effects of defects, impurities, and strain engineering on bandgap modulation, highlighting the necessity of exact control over these parameters. Solving these problems will be essential as the subject develops the electrical properties of borophene for valuable applications.

In addition, the review clarified the environmental elements influencing the stability of borophene, adding to the complexity of real-world applications. Understanding and mitigating these factors will be critical in ensuring the dependability and durability of bromophenol-based gadgets. Despite the current obstacles, the thorough assessment indicates promising directions for further research. By addressing gaps in knowledge and utilizing emerging technology, scientists can create pathways for creative solutions to improve the stability, scalability, and reproducibility of Borophene synthesis by addressing knowledge gaps and utilizing emerging technology. To overcome present obstacles and realize the full potential of borophene in semiconductor technologies, interdisciplinary cooperation and ongoing research into innovative synthesis methods will be crucial. In summary, despite many obstacles to overcome, the possibility of achieving borobopene's technological potential is still exciting. With persistent efforts, the unusual electrical properties of borophene could soon find widespread application in next-generation electronic devices. Additional developments are imminent in the sector.

a) Borophene's excellent performance and controllable features have also made it a promising candidate for use in biological applications such as drug administration, biosensing, and bioimaging. Researchers have synthesized borophene on various metal substrates and investigated its characteristics through elemental doping, defects, and applied mechanical strains. Hydroxy-functionalized borophene production has demonstrated an adjustable band gap and promising prospects for optoelectronic uses. However, large-scale production and the conversion of theoretical and experimental knowledge into useful platforms continue to present difficulties.
b) For several reasons, collaborating on borophene formation theory and experimentation is critical. First, the theoretical study points experimental research in the proper direction by offering insightful information about borophene's characteristics and possible uses. Second, empirical data research validates theoretical predictions and provides crucial information for further theoretical modeling and improvement. This iterative process of collaboration between theory and experiment enables a deeper understanding of the structure, synthesis techniques, and properties of borophene. Researchers may further borophene research and realize its full potential for various applications in energy storage, sensing, and information storage.
c) Suggestions and future paths for expanding the use of borophene in electronics include fostering the development of associated applications such as sensors, energy harvesting, energy storage, energy conversion, and information storage. Researchers should investigate how to scale up the production of graphene-borophene heterostacks while enhancing borophene's electrical characteristics and metallic character. The sonochemical exfoliation approach can make β12 and χ3 Borophene structures, which have demonstrated stability. The liquid-phase exfoliation method is a viable technology for upscaling Borophene manufacturing. Micromechanical exfoliation is one of the innovative methods that has proven effective in producing borophene monolayers and device-quality borophene sheets. Though further study is required to overcome obstacles and make borophene feasible for commercial usage, it has demonstrated potential for storing hydrogen.

## Data availability statement

The dataset supporting the conclusions of this article is included within the article.

## CRediT authorship contribution statement

**Md Abdullah:** Writing – review & editing, Supervision, Data curation, Conceptualization. **Mohammad Saidur Rahman:** Formal analysis, Data curation. **Mohammad Obayedullah:** Writing – original draft, Formal analysis. **Sawda Ahmed Musfika:** Writing – original draft, Formal analysis, Data curation.

## Declaration of competing interest

The authors declare the following financial interests/personal relationships which may be considered as potential competing interests: Md. Abdullah reports writing assistance was provided by City University. Md. Abdullah reports a relationship with City University that includes: non-financial support. Md. Abdullah has patent pending to Md. Abdullah. The authors declare that they have no known competing financial interests or personal relationships that could have appeared to influence the work reported in this paper. If there are other authors, they declare that they have no known competing financial interests or personal relationships that could have appeared to influence the work reported in this paper.
